# The complete chloroplast genome of *Lonicera fulvotomentosa* Hsu et S. C. Cheng and its phylogenetic analysis

**DOI:** 10.1080/23802359.2021.1884027

**Published:** 2021-03-15

**Authors:** Zhengwen Yu, Yin Yi, Lei Gu

**Affiliations:** School of Life Science, Guizhou Normal University, Guiyang, China

**Keywords:** *Lonicera fulvotomentosa*, Caprifoliaceae, complete chloroplast genome, phylogenetic

## Abstract

*Lonicera fulvotomentosa* Hsu et S. C. Cheng is widely used as an edible and medicinal food in China and also displays excellent pharmacological activities. The phylogenetic relationship between *L. fulvotomentosa* and other family members remains unclear. In this work, we assembled the cp genome of *L. fulvotomentosa* using the high-throughput Illumina pair-end sequencing data. The circular cp genome is 155,102 bp in size, including a large single-copy (LSC) region of 88,906 bp and a small single-copy (SSC) region of 18,628 bp, which were separated by two inverted repeat (IR) regions (23,784 bp each). A total of 129 genes were predicted, including eight ribosomal RNAs (rRNAs), 39 transfer RNAs (tRNAs), and 82 protein-coding genes (PCGs). Furthermore, phylogenetic analysis revealed that *L. fulvotomentosa* formed a different clade from other two congeneric species (*Lonicera confuse* and *Lonicera japonica*). This study provides useful information for future genetic study of *L. fulvotomentosa*.

*Lonicera fulvotomentosa* Hsu et S. C. Cheng belongs to the Caprifoliaceae family, is distributed in mountainous areas of southern China (northwest of Guangxi, southwest of Guizhou and Yunnan). It also has a commonly known Chinese name as ‘Shan Yin Hua’. To date, many active ingredients, including caffeoylquinic acid, cerebrosides, nitrogen-containing iridoid glycosides, and triterpene glycosides, have been isolated and characterized from *Lonicera* species (Peng et al. 2000; Lin et al. 2008; Zheng et al. 2012; Yu et al. 2015; Kong et al. 2017). These active ingredients have shown anti-allergic, anti-inflammatory, anti-bacterial, and anti-viral activities (Xu et al. 2007; Xiong et al. 2013). Although *L. fulvotomentosa* shows economic value, few genetic researches have been done on this plant. In this work, the complete chloroplast (cp) genome of *L. fulvotomentosa* has been reported, the assembled and determined cp genome sequence of *L. fulvotomentosa* will be a useful resource for future genetic and genomic research.

Young and healthy leaf samples were collected from Dashuijing Village, Dewo Town, Anlong County, Guizhou Province, China (25°0′53.57″N, 105°14′17.73″E, 1141 m above sea level). The leaf specimen (accession number: GZNUYZW202003001) was deposited in the herbarium of School of Life Sciences, Guizhou Normal University. The total genomic DNA (no. YZW202003002) was extracted using DNAsecure Plant Kit (TIANGEN, Beijing, China) and stored at −80 °C in the laboratory (room number: 1403) of School of Life Science, Guizhou Normal University. A total amount of 700 ng DNA per sample was used as input material for the DNA sample preparations. Sequencing libraries were generated using NEB Next^®^ Ultra DNA Library Prep Kit for Illumina^®^ (NEB, Ipswich, MA). Total DNA was used to generate libraries with an average insert size of 350 bp. The library preparations were sequenced on an Illumina platform and 150 bp paired-end reads were generated. The filtered reads were assembled using the program GetOrganelle (Jin et al. 2019) with *Lonicera japonica* (GenBank accession number: MH028738) as the initial reference genome. The assembled cp genome was annotated using the online software GeSeq (Tillich et al. 2017). The accurate annotated complete cp genome was submitted to GenBank with accession number MW186760.

The length of the complete cp genome sequence of *L. fulvotomentosa* is 155,102 bp, consisting of a large single-copy (LSC, 88,906 bp) region, a small single-copy (SSC, 18,628 bp) region, and two inverted repeat (IRA and IRB) regions of 23,784 bp each. Totally, 129 genes were predicted, including 82 protein-coding genes (PCGs), eight ribosomal RNA (rRNA) genes, and 39 transfer RNA (tRNA) genes. Among these assembled genes, all rRNAs, five PCGs (*rps7*, *rpl12*, *ndhB*, *ycf2*, and *ycf15*) and seven tRNAs (*trnA-UGC*, *trnI-CAU*, *trnI-GAU*, *trnL-CAA*, *trnN-GUU*, *trnR-ACG*, and *trnV-GAC*) were with double copies. One tRNA (*trnG-GCC*) occurs in three copies. Intron-exon analysis showed the majority (105 genes, 81%) genes with no introns, whereas 24 (19%) genes contain introns.

To further understand the cp genome of *L. fulvotomentosa*, 20 cp genome sequences of Caprifoliaceae family (12 *Lonicera* species, two species from genus *Dipelta*, one *Heptacodium* species, one specie from genus *Triosteum*, one *Weigela* species, and three species from genus *Patrinia*) were downloaded from GenBank to construct the phylogenetic trees through maximum-likelihood (ML) analysis. The ML tree was performed using RAxML (Version 8.0.19, GTRGAMMA) (Stamatakis 2014) with 1000 bootstrap replicates. The phylogenetic tree indicated that *L. fulvotomentosa* belongs to genus *Lonicera* (Figure 1) and formed a different clade from *Lonicera confuse* and *Lonicera japonica* (Figure 1).

**Figure 1.**
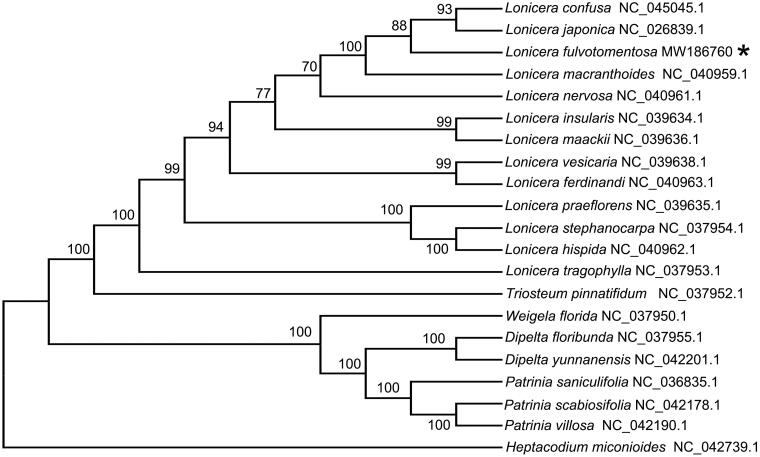
Maximum-likelihood tree based on the complete cp genome sequences of 20 species from the Caprifoliaceae family. GenBank accession numbers are described in the figure. Shown next to the nodes are bootstrap support values based on 1000 replicates.

## Data Availability

The annotated chloroplast genome data that support the findings of this study are openly available in GenBank of NCBI at https://www.ncbi.nlm.nih.gov under the accession number MW186760. The associated BioProject, SRA, and Bio-Sample numbers are PRJNA674956, SRX9460984, and SAMN16684233, respectively.
